# Percutaneous kyphoplasty for osteoporotic vertebral compression fractures performed in one-day surgery: safe and effective?

**DOI:** 10.3389/fsurg.2025.1636150

**Published:** 2025-10-10

**Authors:** Zhongcheng An, Jiayi Dou, Wangnan Mao, Bing Wu, Han Zhang, Junwei Feng, Chen Chen, Binbin Tang, Liqiang Dong, Lianguo Wu, Xiaoping Zhang

**Affiliations:** 1 Department of Orthopedics and Traumatology, the Second Affiliated Hospital of Zhejiang Chinese Medical University, Hangzhou, Zhejiang, China; 2 The Second Clinical Medical College, Zhejiang Chinese Medical University, Hangzhou, Zhejiang, China; 3 Department of Geriatrics, The Second Affiliated Hospital of Zhejiang Chinese Medical University, Hangzhou, China

**Keywords:** percutaneous kyphoplasty, osteoporosis vertebral compression fractures, day surgery, delayed discharge, spine

## Abstract

**Objective:**

Evaluating the safety and efficacy of percutaneous kyphoplasty (PKP) as an ambulatory surgery procedure, and analyzing causes of postoperative delayed discharge in day surgery settings.

**Methods:**

A retrospective analysis was conducted on 299 patients diagnosed with osteoporotic vertebral compression fractures (OVCFs) who required PKP surgery in our hospital's orthopedic department between January 2022 and December 2023. Among them, 157 underwent the day surgery procedure group (DSP), while 142 received the traditional inpatient group (TIP). The following parameters were recorded for both groups: gender, age, preoperative comorbidities, fracture location, preoperative bone density T-score, preoperative ASA classification, operative time, intraoperative blood loss, cement leakage, pulmonary embolism, bone cement toxicity, cement injection volume, puncture site hematoma, infection, cerebrospinal fluid leakage, 1-month postoperative refracture incidence, Visual Analogue Scale (VAS) scores and Oswestry Disability Index (ODI) scores at preoperative, postoperative day 1, and 1-month postoperative timepoints, along with discharge satisfaction.

**Results:**

No statistically significant differences were observed between the two groups in age, gender, bone mineral density, fracture level distribution, ASA classification, operative time, intraoperative blood loss, cement leakage incidence, or cement injection volume (*P* > 0.05). In terms of comparing surgical efficacy, there was no statistically significant difference in VAS score and ODI score between the two groups of patients before surgery, 1 day after surgery and 1 month after surgery (*P* > 0.05). The DSP group showed significantly shorter hospitalization (0.95 ± 0.28 days vs. TIP: 5.20 ± 1.37 days, *P* < 0.05) and lower hospitalization costs (22,056.66 ± 2,337.61 CNY vs. TIP: 28,341.12 ± 1,711.45 CNY, *P* < 0.05). Patient satisfaction was significantly higher in the DSP group (96.39 ± 2.26 vs. TIP: 93.87 ± 2.28, *P* < 0.05), attributable to reduced hospitalization duration and costs. Among 157 day surgery patients, 133 successfully completed the day surgery pathway, while 24 required conversion to traditional inpatient care (DSPT). No significant differences existed in preoperative or 1-month postoperative VAS/ODI scores between DSP and DSPT subgroups (*P* > 0.05). However, at postoperative day 1, the DSP subgroup demonstrated superior VAS and ODI scores compared to DSPT (*P* < 0.05).

**Conclusions:**

PKP demonstrates safety and efficacy as an day surgery for OVCF, warranting widespread adoption. However, clinicians should note that suboptimal pain relief on postoperative day 1 may represent a primary factor contributing to delayed discharge in some patients.

## 1 Introduction

With the accelerated aging of the population, osteoporotic vertebral compression fracture (OVCF) has become a prevalent condition among older adults. Its rising incidence significantly compromises patients' quality of life, thereby garnering increasing clinical attention (1, 2). OVCFs often cause acute/chronic back pain, spinal deformity, functional disability, and reduced quality of life, while being associated with a 5-year mortality rate exceeding that of hip fractures (3–5).

In recent years, percutaneous kyphoplasty (PKP) has become the most commonly used minimally invasive method for the treatment of OVCFs. It is definitely safe and short-term efficacious (6–8), allowing patients to get out of bed early for rehabilitation exercises and effectively avoiding serious complications associated with vertebral fractures. However, most published studies on PKP for OVCFs report mean hospitalization durations of 5–15 days. Such prolonged hospitalization imposes considerable patient inconvenience and elevated healthcare costs (9–11).

As a new mode typified by 24-hour discharge, day surgery has developed rapidly in China and internationally in the past 20 years. PKP, which is correct with short times, less traumatic, less bleeding, less disturbance to the surrounding tissue neural structures, and is performed under local anesthesia. Therefore, it has the basis to become a one-day surgery for patients with OVCFs, with the characteristics of saving medical resources, safety and efficiency, and convenience and efficiency (12). However, some patients may have poor postoperative outcomes, which seriously affects the efficacy of the operation and patient satisfaction, resulting in delayed discharge from the hospital (13). Therefore, this study aims to validate the safety and efficacy of PKP as an ambulatory surgery for OVCF through clinical trials, while exploring potential factors contributing to delayed discharge.

## 2 Materials and methods

### 2.1 Patients

A total of 299 patients diagnosed with osteoporotic vertebral compression fractures (OVCFs) scheduled for PKP were enrolled in the Department of Orthopedics between January 2022 and December 2023. Based on patient preference, participants were allocated to either the day surgery group (DSP, *n* = 157) or the traditional inpatient group (TIP, *n* = 142). The DSP cohort comprised 58 males and 99 females aged 60–75 years (mean 67.29 ± 4.50). Fracture distribution included: thoracic (*n* = 23), thoracolumbar (*n* = 79), and lumbar (*n* = 55) levels. ASA classifications were: Grade I (*n* = 53), II (*n* = 76), and III (*n* = 28). The TIP cohort included 52 males and 90 females aged 60–80 years (mean 67.29 ± 4.50). Fracture distribution comprised: thoracic (*n* = 19), thoracolumbar (*n* = 71), and lumbar (*n* = 52) levels. ASA classifications were: Grade I (*n* = 43), II (*n* = 72), and III (*n* = 27).

This study received ethical approval from the Institutional Review Board of The Second Affiliated Hospital of Zhejiang Chinese Medical University (Approval No. 2021-KL-062-01). Written informed consent was obtained from all participants prior to surgery.

### 2.2 Selection criteria

Inclusion criteria: (1) Aged patients diagnosed with single-level osteoporotic vertebral compression fractures (involving the thoracic or lumbar spine), confirmed through medical history, clinical symptoms, physical examination, and comprehensive imaging studies; (2) CT confirmation of vertebral body height loss <66% with preserved posterior wall integrity; (3) American Society of Anesthesiologist (ASA) classification ≤ III without significant cardiopulmonary comorbidities; (4) Demonstrated treatment adherence and absence of major communication barriers; (5) Informed consent for the one-day surgery protocol after comprehensive understanding of benefits/risks, with willingness for potential conversion to inpatient care per patient preference.(6)All patients were classified according to the DGOU criteria (OF1 and OF2 types) and met the criteria for minimally invasive PKP surgery.

Exclusion criteria: (1) pathological fractures, including tumors, infections, etc.(2) vertebral burst fracture or compression fractures with spinal canal stenosis and nerve injury symptoms.(3) patients with cognitive impairment that precluded informed consent or understanding of postoperative instructions; (4) patients with cardiopulmonary disorders and other organ complications, ASA score is more than III;(5) multi-segment vertebral compression fracture.

### 2.3 Data collection

Relevant literature and clinical experience were used to determine the related factors that may affect the delayed discharge of OVCFs day surgery. The following data for all patients were collected: gender, age, preoperative comorbidities, fracture level, preoperative bone mineral density(BMD) T-scores, preoperative ASA score, operative duration, intraoperative blood loss, bone cement leakage, pulmonary embolism, bone cement toxic reaction, the amount of cement injected, puncture site hematoma, infection, cerebrospinal fluid leakage, the occurrence of re-fracture 1 month after operation, Visual Analogue Scale(VAS) and Oswestry Disability Index(ODI) before operation,1 day and 1 month after operation, patients satisfaction at discharge. [The above data are based on a percentage-based grading system (0–100)].

### 2.4 Treatment method

**Figure 1:**
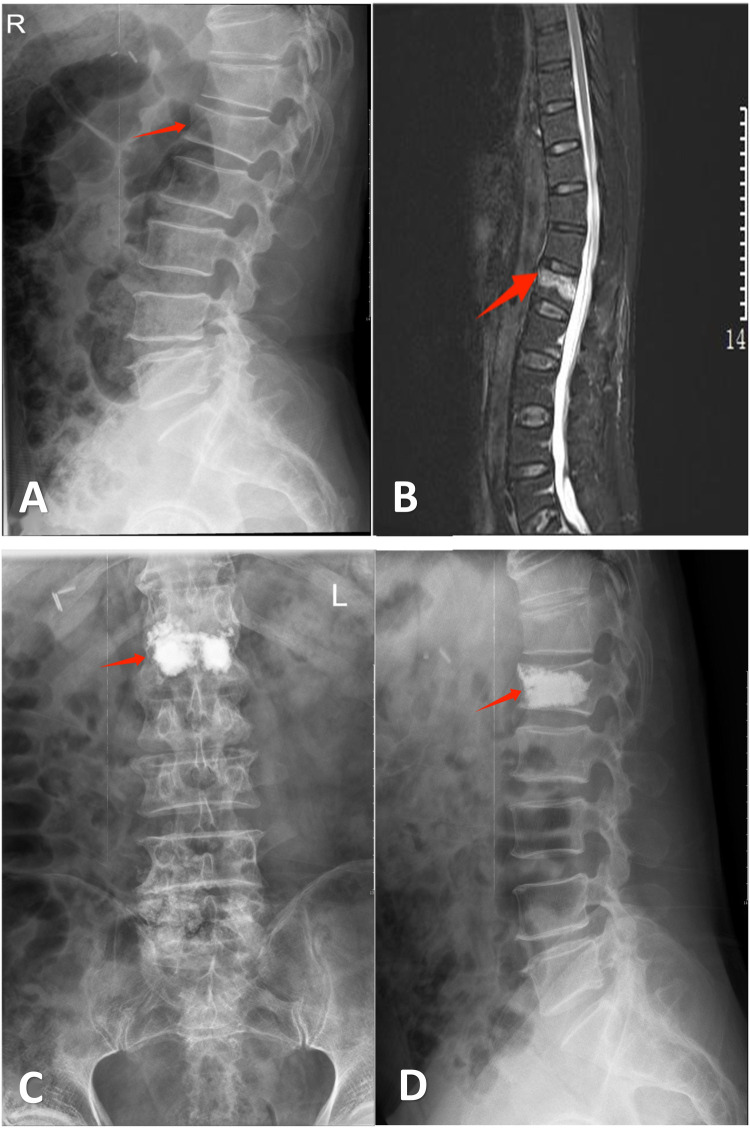
Mr. Hu, a 74-year-old male, was admitted to our hospital due to “low back pain for 1 day following trauma”. Diagnosis: L1 vertebral compression fracture. He underwent bilateral percutaneous kyphoplasty (PKP). Persistent postoperative pain on day 1 precluded same-day discharge, necessitating transfer to the conventional treatment group. **(A)** Lateral radiograph demonstrating wedging deformity of the L1 vertebral body. **(B)** Sagittal MRI revealing an L1 vertebral fracture. **(C,D)** Postoperative anteroposterior and lateral radiographs after bilateral PKP at L1.

**Figure 2:**
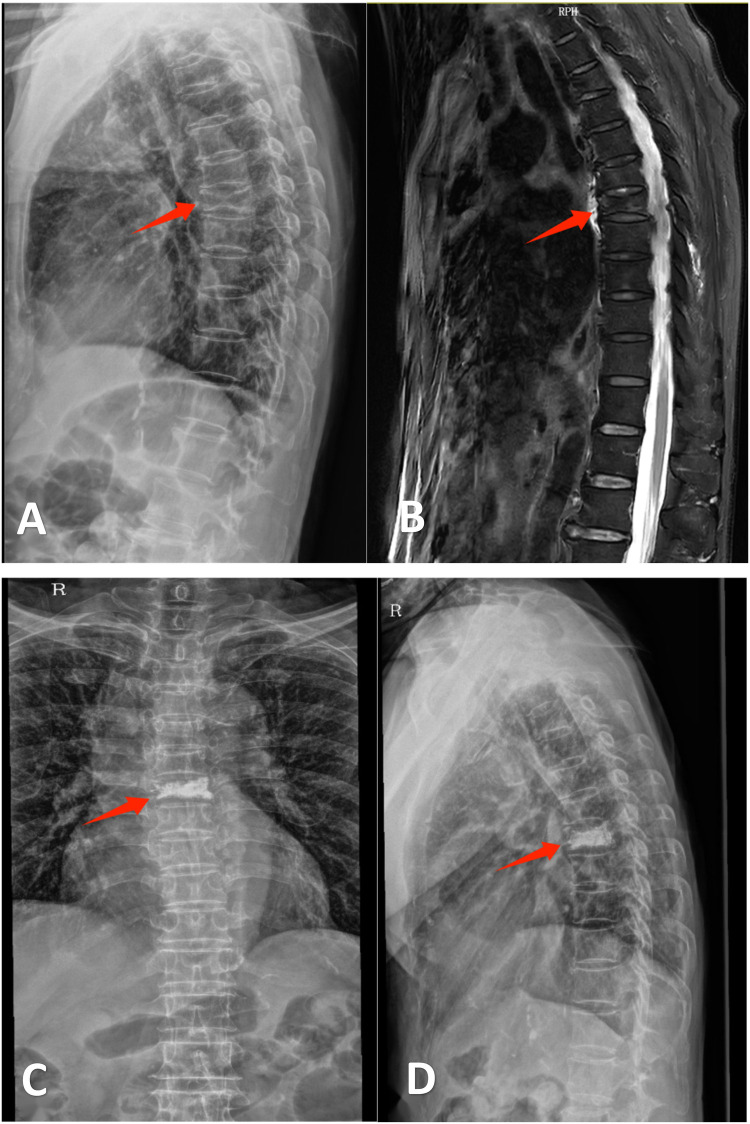
Ms. Zhou, a 76-year-old female, was admitted for “thoracic back pain persisting for 4 days following a twisting injury.” Diagnosis: T6 vertebral compression fracture. She underwent unilateral percutaneous kyphoplasty (PKP). Postoperative pain was significantly alleviated, thus permitting same-day discharge. **(A)** Lateral radiograph demonstrating wedging deformity of the T6 vertebral body. **(B)** Sagittal MRI revealing a T6 vertebral fracture. **(C,D)** Postoperative anteroposterior and lateral radiographs after unilateral PKP at T6.

For DSP group patients, a definitive diagnosis was established during outpatient evaluation with the completion of all preoperative imaging and laboratory tests. One-day admission was arranged for surgical intervention on the day of hospitalization. TIP group patients underwent preoperative workup after hospital admission. Both cohorts underwent PKP procedures under local anesthesia with monitored anesthesia care (MAC), following standardized surgical protocols (14) (Figures 1, 2).

The brief operation process of PKP surgery is as follows: (1) C-arm fluoroscopy is utilized to identify the targeted vertebral segments. A skin mark is placed at the surface projection of the intended puncture site. Following successful local anesthesia with 0.1% lidocaine, percutaneous puncture is initiated at the marked location. (2) Under continuous C-arm fluoroscopic guidance, the puncture needle is advanced into the fractured vertebra. Once proper placement is confirmed (and balloon kyphoplasty is performed for vertebral body reduction if necessary), bone cement is prepared. The cement is then injected into the vertebra using a push rod, with real-time fluoroscopic monitoring to assess cement distribution within the vertebral body. (3) The bone cement typically requires approximately 15 min to fully polymerize. Upon complete hardening of the cement, the puncture needle is carefully withdrawn, manual pressure is applied to achieve hemostasis, and the wound is dressed. The procedure is then considered complete.

Patients returned to the ward for bed rest and were allowed to eat and drink after the operation. Patients were able to ambulate with a lumbar brace at 2 h postoperatively. Depending on the patient's condition, analgesics would be given as required if patients had surgical site pain. The patient was discharged on the following day after an overnight observation in the ward if they met the criteria for day surgery discharge. Patients experiencing uncontrolled clinical deterioration must be immediately converted from the ambulatory pathway to inpatient care, with multidisciplinary consultations initiated when necessary. For the TIP group, discharge timing was determined by patient preference. Upon discharge, all patients received standardized anti-osteoporosis pharmacotherapy: Calcitriol 0.25 μg twice daily, Caltrate 600 mg once daily, and Alendronate Sodium 70 mg weekly. This regimen was supplemented with physical therapy, structured rehabilitation exercises, and prescribed kinesitherapy.

### 2.5 Discharge criteria

Following morning ward rounds on postoperative day 1, the following assessments were conducted: ODI and VAS scoring, efficacy and satisfaction evaluations, and radiographic re-examination. The discharge criteria for day surgery in our department are as follows: (1) post-anesthesia discharge score(PADS) ≥ 9, all vital signs were stable, and there was no obvious swelling and bleeding from the wound; (2) VAS score is less than 3 points; (3) Complications of surgery that do not require further management, or symptoms that resolve significantly with rest; (4) No significant discomfort after eating and drinking, normal urination and defecation, patients can walk around by themselves; (5) The patient received bedside care and assistance post-discharge.

### 2.6 Statistical analyses

Collected data were analyzed using SPSS 22.0 statistical software (SPSS, Inc., Chicago, IL, USA). Quantitative data were shown as the `x ± s, and qualitative data were expressed as the frequency (%). Quantitative data were compared with the independent-sample *T*-test. Qualitative data were compared with the x^2^ test. At the same time, the ANOVA was performed to assess the differences within groups. Data that did not meet normal distribution were presented with medians(interquartile) [M (Q1, Q3)]. *P* < 0.05 indicated that the difference was statistically significant.

## 3 Result

All 299 patients successfully completed the surgical procedure. Among the 157 DSP cohort patients, 133 (84.7%) completed the ambulatory pathway as planned, while 24 (15.29%) proceeded to the DSPT subgroup following conversion to inpatient care.

### 3.1 Patient demographic data of two groups (DSP and TIP)

No statistically significant differences in age, gender, BMD, fracture level, and ASA score were found between the two groups(*P* > 0.05) (Table 1).

**Table 1:** Patient demographic data of both groups (DSP and TIP).

Group	General information			Fracture level			ASA physical status classes		
	Age (years)	Gender (male/female)	BMD	Thoracic(T4-T10)	Thoracolumbar (T11-L2)	Lumbar(L3-L5)	ASA I	ASA II	ASA III
DSP group (*n* = 133)	68.05 ± 4.49	48/85	−2.77 ± 0.81	21	69	43	49	64	20
TIP group(*n* = 142)	69.15.79 ± 5.66	52/90	−2.92 ± 0.80	19	71	52	43	72	27
*P* value	0.074	0.927	0.127	0.927	0.927	0.927	0.447	0.447	0.447

### 3.2 Occurrence of complications in two groups (DSP and TIP)

No statistically significant differences were observed between groups in operative time, intraoperative blood loss, cement leakage incidence, or cement injection volume (*P* > 0.05). Two patients developed localized hematomas at the needle insertion site, causing procedure-related back pain that necessitated conversion to inpatient care. Other complications—including pulmonary embolism, bone cement systemic reactions, surgical site infection, and 1-month postoperative refractures—were not observed in either cohort (Table 2).

**Table 2:** Postoperative complications of both groups (DSP and TIP).

Group	Operative duration (min)	Intraoperative blood loss (ml)	Cement leakage (n)	The amount of cement injected
DSP group(*n* = 133)	47.71 ± 10.50	4.93 ± 1.37	7	4.14 ± 1.40
TIP group(*n* = 142)	48.51 ± 10.11	4.85 ± 1.44	7	3.94 ± 1.49
*P* value	0.520	0.637	0.900	0.274

### 3.3 ODI scores and VAS scores of two groups (DSP and TIP)

The postoperative ODI scores and VAS scores in both two groups were dramatically improved compared with the preoperative period, and the differences were statistically significant (*P* < 0.05); There was no significant difference in VAS scores and ODI scores between the two groups of patients before the operation, 1 day after the operation, and 1 month after the operation. (*P* > 0.05) (Table 3).

**Table 3:** Comparison of Oswestry Disability Index (ODI) scores and VAS scores in two groups (DSP and TIP).

Group	VAS scores			ODI scores		
	Preoperative period	1 day after operation	1 month after operation	Preoperative period	1 day after operation	1 month after operation
DSP (*n* = 133)	5.56 ± 1.11	1.86 ± 0.78*	0.89 ± 0.81*	35.97 ± 3.94	14.78 ± 2.02*	7.44 ± 1.69*
TIP (*n* = 142)	5.54 ± 1.10	1.95 ± 0.82*	0.96 ± 0.80*	36.39 ± 3.78	15.09 ± 1.97*	7.65 ± 1.66*
*T* value	0.159	−0.893	−0.796	−0.897	−1.287	−1.045
*P* value	0.874	0.373	0.427	0.371	0.199	0.297

Sig: This symbol (*) indicates that the difference is statistically significant compared with that before the operation. (P < 0.05).

### 3.4 Hospital costs, hospital stay and satisfaction of two groups (DSP and TIP)

The hospital stay duration of the DSP group (0.95 ± 0.28 days) was significantly shorter than that of the TIP group (5.20 ± 1. 37 days), the differences were statistically significant (*P* < 0.05). In terms of hospital costs, the DSP group (22,056.66 ± 2,337.61 CNY) showed significantly lower than the TIP group (28,341.12 ± 1,711.45 CNY, *P* < 0.05). For patient satisfaction, patients in group DSP had higher satisfaction because of lower hospital costs and shorter hospital stays (DSP: 96.39 ± 2.26 vs. TIP: 93.87 ± 2.28), the differences were statistically significant (*P* < 0.05). (Table 4).

**Table 4:** Comparison of the hospital stay, hospital costs and satisfaction between the two groups (DSP and TIP).

Group	Hospital stay(days)	Hospital costs(CNY)	Patient satisfaction
DSP (*n* = 133)	0.95 ± 0.28	22,056.66 ± 2,337.61	96.39 ± 2.26
TIP(*n* = 142)	5.20 ± 1. 37	28,341.12 ± 1,711.45	93.87 ± 2.28
*T* value	−35.164	−25.550	9.176
*P* value	<0.001	<0.001	<0.001

### 3.5 ODI scores and VAS scores of two groups (DSP and DSPT)

Within-group comparisons demonstrated significant improvement in both VAS and ODI scores from preoperative to postoperative assessments for DSP and DSPT subgroups (*P* < 0.05). No significant inter-subgroup differences existed in preoperative or 1-month postoperative scores (*P* > 0.05). However, at postoperative day 1, the DSP cohort showed superior VAS and ODI scores compared to DSPT, with statistically significant differences (*P* < 0.05). (Table 5).

**Table 5:** Comparison of oswestry disability Index (ODI) scores and VAS scores in two groups (DSP and DSPT).

Group	VAS scores			ODI scores		
	Preoperative period	1 day after operation	1 month after operation	Preoperative period	1 day after operation	1 month after operation
DSP (*n* = 133)	5.56 ± 1.11	1.86 ± 0.78*	0.89 ± 0.81*	35.97 ± 3.94	14.78 ± 2.02*	7.44 ± 1.69*
DSPT(*n* = 24)	5.42 ± 1.21	2.33 ± 0.64*	1.00 ± 0.89*	36.04 ± 4.03	15.96 ± 1.55*	8.04 ± 1.76*
*T* value	0.559	−2.790	−0.617	−0.082	−2.715	−1.583
*P* value	0.577	0.006	0.538	0.935	0.007	0.115

Sig: This symbol (*) indicates that the difference is statistically significant compared with that before the operation. (*P* < 0.05).

## 4 Discussion

As demonstrated in our cohort, patients with OVCFs are typically elderly (mean age: 67.29 ± 4.5 years), with a high prevalence of comorbidities. The theory of early treatment of fractures holds that early treatment can reduce the occurrence of complications such as pneumonia and deep vein thrombosis after fracture, and reduce the burden of family care. Early treatment of fractures also reduces hospital stay and costs, reduces the risk of nosocomial infection, and Improve the efficiency of diagnosis and treatment (15). As the most commonly used minimally invasive surgical therapy for treating OVCFs, PKP has become the most accepted treatment modality for patients due to easy surgical operation, significant symptom relief, and few complications. Meanwhile, day surgery with shorter hospital stays, lower risk of infection, faster recovery, and lower cost has become more popular among clinicians and patients. Therefore, it is reasonable to assume that PKP treatment for day surgery procedures is feasible.

Our comparative analysis of 133 day surgery patients and 142 traditional inpatients revealed no significant intergroup differences in age, gender, bone mineral density, fracture level distribution, or ASA classification (*P* > 0.05). Complication rates during and after surgery were comparable between cohorts (*P* > 0.05). Both groups demonstrated significant improvements in VAS and ODI scores postoperatively vs. preoperative baselines (all *P* < 0.05), indicating that discharge within 24 h following PKP is safe and effective. Zhang et al. (16) found comparable efficacy and safety between day surgery vertebroplasty and conventional hospitalization for OVCFs during follow-up of 159 patients for greater than or equal to 1 year. Nie et al. (12) believe that the use of PVP to treat OVCFs in the daytime mode is safe, effective, and worthy of promotion. Therefore, we conclude that ambulatory management provides a viable alternative to traditional hospitalization for most OVCF patients, demonstrating equivalent safety and efficacy profiles.

In this study, initially, 157 patients adopted the day surgery mode, but ultimately only 133 completed the day surgery. 24 patients were transferred to traditional hospitalization (DSPT group) due to reasons such as pain not meeting the discharge criteria, patients' anxiety, and no care at home for older patients. The completion rate of day surgery was 84.71% (133/157). Due to the short hospital stay in the day surgery mode, many patients are concerned about the uncertainty of day surgery, especially in terms of postoperative recovery. Some people still choose to rest continuously in the hospital after the operation, believing that they recover better under the direct supervision of doctors. This is often contrary to the original intention of day surgery (17). In this group of patients, the majority did not express a concern about the one-day hospital stay. Two of them experienced lower back pain due to hematoma at the puncture site, while more patients felt that their pain relief did not meet the ideal expectations on the first day after the operation. Therefore, in terms of VAS and ODI scores, the VAS score and ODI score of the DSP group on the first day after surgery were lower than those of the DSPT group (*P* < 0.05), but at 1 month after surgery, there was no significant difference in VAS score and ODI score between the two groups of patients. This indicates that residual low back pain after PKP may be an important reason for patients' inability to complete the one-day mode. Our team's previous research found that most of the early postoperative residual low back pain was caused by low back fascia injury, intraoperative puncture, and other reasons, which would have a negative impact on the short-term efficacy and satisfaction of patients (18). YAO et al. (19) also hold that posterior fascia edema and puncture injury of small joints during the operation are the main causes of postoperative residual low back pain. Most meta-analyses of studies have confirmed that the clinical efficacy of unilateral puncture is comparable to that of bilateral puncture, but it can reduce back puncture injury. Therefore, some scholars suggest that unilateral puncture should be chosen as much as possible during PKP surgery (20–22). Therefore, we believe that the main reasons for the significant injury of the back muscles and fascia caused by the severe trauma of the patients before the operation or the puncture injury during the operation leading to the pain of the patients on the first day after the operation may be the important factors causing the patients to be unable to complete the daytime mode.

In this study, the hospital stay of the DSP group was significantly shorter than that of the TIP group (*P* < 0.05), and the hospital cost of DSP was lower than the TIP group (*P* < 0.05), and the DSP group had higher patient satisfaction (*P* < 0.05). This may be related to the whole treatment process in the hospital. For regular inpatients, they need to wait for a bed before hospitalization. After hospitalization, they need to complete preoperative examinations and wait for the surgeon's operation day to perform the surgery. For patients undergoing day surgery, the examination is generally completed preoperatively, and because of the outpatient efficient rate, the preoperative examination can also be completed relatively quickly with no need to wait after hospitalization. Under the guarantee of an efficient and rapid turnover of the Department, adequate beds and experienced surgeons are available every day to perform the surgery for the patients. In China, where social health resources are limited, shorter hospital stays and less hospital expenditure for day surgery could reduce the insurance burden and the financial burden on patients and improve patient satisfaction.

Certainly, our study has some limitations. First and foremost, this study is inherently limited by its retrospective design, which increases susceptibility to various biases. Secondly, the patients we included in day surgery had fewer comorbidities and could not fully cover all patients with osteoporotic spinal fractures. Thirdly, due to the relatively small number of people converted from day surgery to regular hospitalization, the relatively small population limits the statistical power of the study and may lead to statistical bias in the causes of delayed discharge from day surgery. Therefore, further studies with multiple centers and large samples are needed. Fourth, the age of our research participants was relatively young. In clinical practice, some patients over 80 years old have more comorbidities. How to include these patients in day surgery is the direction of our future efforts. At present, day surgery in China is still in its infancy and it cannot blindly apply foreign models. It is necessary to establish a standardized day surgery model suitable for the local medical structure and level based on the basic national conditions and the medical levels of various regions. It should be particularly noted that although this study focuses on the surgical treatment of confirmed fractures, the prevention of fractures remains the top priority. In the future, the prevention of fractures in China will first involve early screening and diagnosis. Secondly, during the treatment process, attention should be paid to the application of anti-osteoporosis drugs. Thirdly, measures to prevent falls among the elderly should be taken, which is crucial for reducing the overall incidence of OVCFs.

## 5 Conclusion

Overall, compared with traditional inpatient procedures, performing PKP as one-day surgery procedure may decrease the duration of hospital stay, minimize hospital costs, and improve patient satisfaction, and its safety and efficacy were comparable to those of traditional inpatient procedures. Therefore, we suggest that the application of PKP in the treatment of OVCFs under the mode of one-day surgery can be effective in reducing the hospital length of stay and procedure expenditure. However, it should be noted that when the patients' pain is not relieved on the first day after the operation, they can be subject to a delayed discharge.

## Data Availability

The original contributions presented in the study are included in the article/Supplementary Material, further inquiries can be directed to the corresponding author.

## References

[1] Zhu HT Ding DG Wang S Zhu YL. Comparison between percutaneous kyphoplasty and percutaneous vertebroplasty in terms of efficacy in osteoporotic vertebral compression fractures: a meta-analysis. Altern Ther Health Med. (2022) 28(5):49–53. Available online at: https://pubmed.ncbi.nlm.nih.gov/35648693/35648693

[2] Li WS Cai YF Cong L. The effect of vertebral augmentation procedure on painful OVCFs: a meta-analysis of randomized controlled trials. Global Spine J. (2022) 12(3):515–25. 10.1177/2192568221999369 33706568 PMC9121160

[3] Liu X Liu H Dong Y Yang X Zou J Ren L Protocol for systematic review and meta-analysis on the efficacy and safety of acupuncture for residual low back pain after percutaneous kyphoplasty in patients with osteoporotic vertebral compression fracture. BMJ Open. (2024) 14(8):e082272. 10.1136/bmjopen-2023-082272 39209779 PMC11404181

[4] Zhuang M Cai B Wang F. Effectiveness and safety of percutaneous kyphoplasty combined with zoledronic acid in treatment of osteoporotic vertebral compression fractures: a meta-analysis. Arch Orthop Trauma Surg. (2022) 142(10):2435–43. 10.1007/s00402-021-03858-4 33713186

[5] Rizkallah M Bachour F Khoury ME Sebaaly A Finianos B El Hage R Comparison of morbidity and mortality of hip and vertebral fragility fractures: which one has the highest burden? Osteoporos Sarcopenia. (2020) 6(3):146–50. 10.1016/j.afos.2020.07.002 33102809 PMC7573502

[6] Láinez Ramos-Bossini AJ López Zúñiga D Ruiz Santiago F. Percutaneous vertebroplasty versus conservative treatment and placebo in osteoporotic vertebral fractures: meta-analysis and critical review of the literature. Eur Radiol. (2021) 31(11):8542–53. 10.1007/s00330-021-08018-1 33963449

[7] Sanli I van Kuijk SMJ de Bie RA van Rhijn LW Willems PC. Percutaneous cement augmentation in the treatment of osteoporotic vertebral fractures (OVFs) in the elderly: a systematic review. Eur Spine J. (2020) 29(7):1553–72. 10.1007/s00586-020-06391-x 32240375

[8] Liu D Xu J Wang Q Zhang L Yin S Qian B Timing of percutaneous balloon kyphoplasty for osteoporotic vertebral compression fractures. Pain Physician. (2023) 26(3):231–43. 10.36076/ppj.2023.26.231 37192225

[9] Liu Z Li H Tang Y Liu H Zhang J Zou J Comparison of unilateral and bilateral percutaneous kyphoplasty for the treatment of osteoporotic vertebral compression fractures associated with scoliosis. Exp Ther Med. (2023) 26(1):335. 10.3892/etm.2023.12034 37383374 PMC10294595

[10] Luo K Jiang G Zhu J Lu B Lu J Zhang K Poly(methyl methacrylate) bone cement composited with mineralized collagen for osteoporotic vertebral compression fractures in extremely old patients. Regen Biomater. (2020) 7(1):29–34. 10.1093/rb/rbz045 32153989 PMC7053255

[11] Ma M Wang Z Ye J Chen X. Effectiveness of TiRobot-assisted and free-hand percutaneous kyphoplasty via pedicle approach in the treatment of osteoporotic vertebral compression fractures of the thoracic vertebra. Chin J Reparative Reconstr Surg. (2023) 37(9):1106–12. 10.7507/1002-1892.202305035 PMC10505635 37718423

[12] Nie B Wang Q Li B Ou N Yang Z. Exploration of percutaneous vertebroplasty in the treatment of osteoporotic vertebral compression fracture as day surgery: a retrospective study. Eur Spine J. (2021) 30(9):2718–25. 10.1007/s00586-021-06887-0 34075472

[13] An Z Chen C Wang J Zhu Y Dong L Wei H Logistic regression analysis on risk factors of augmented vertebra recompression after percutaneous vertebral augmentation. J Orthop Surg Res. (2021) 16(1):374. 10.1186/s13018-021-02480-9 34116683 PMC8194186

[14] Margetis K Patel A Petrone B Carter KR. Percutaneous vertebroplasty and kyphoplasty. In: Statpearls [Internet]. Treasure Island (FL): StatPearls Publishing (2025). PMID: 30247838 30247838

[15] Prost S Pesenti S Fuentes S Tropiano P Blondel B. Treatment of osteoporotic vertebral fractures. Orthop Traumatol Surg Res. (2021) 107(1S):102779. 10.1016/j.otsr.2020.102779 33321233

[16] Zhang QW Sun CT Wang Q. Evaluation of the efficacy and safety of vertebroplasty as a day surgery procedure. Chin J Geriatr. (2020) 39(11):1318–22. 10.3760/cma.j.issn.0254-9026.2020.11.017

[17] Rodriguez LV Bloomstone JA. Benchmarking outcomes for day surgery. Best Pract Res Clin Anaesthesiol. (2023) 37(3):331–42. 10.1016/j.bpa.2023.03.001 37938080

[18] Chen C An ZC Wu LG Pang ZD Xiao LG Wei H Analysis of the causes of residual back pain in the early and late stages after percutaneous vertebral augmentation. Zhongguo Gu Shang. (2022) 35(8):724–31. 10.12200/j.issn.1003-0034.2022.08.005 35979764

[19] Li Y Yue J Huang M Lin J Huang C Chen J Risk factors for postoperative residual back pain after percutaneous kyphoplasty for osteoporotic vertebral compression fractures. Eur Spine J. (2020) 29(10):2568–75. 10.1007/s00586-020-06493-6 32507918

[20] Sun Y Li X Ma S Chong H Cai TC Li KM Comparison of the efficacy and safety of unilateral and bilateral approach kyphoplasty in the treatment of osteoporotic vertebral compression fractures: a meta-analysis. Jt Dis Relat Surg. (2024) 35(3):491. 10.52312/jdrs.2024.1701 39189557 PMC11411882

[21] Qiao Y Wang X Liu Y Hu J Yuan FH Zhao ZG. Comparison of unilateral and bilateral percutaneous kyphoplasty for osteoporotic vertebral compression fractures. J Pain Res. (2023) 16:1813–23. 10.2147/JPR.S393333 37273274 PMC10239257

[22] Cao DH Gu WB Zhao HY Hu JL Yuan HF. Advantages of unilateral percutaneous kyphoplasty for osteoporotic vertebral compression fractures—a systematic review and meta-analysis. Arch Osteoporos. (2024) 19:38. 10.1007/s11657-024-01400-8 38750277

